# Novel Thiazolidinone/Thiazolo[3,2-*a*]Benzimidazolone-Isatin Conjugates as Apoptotic Anti-proliferative Agents Towards Breast Cancer: One-Pot Synthesis and In Vitro Biological Evaluation

**DOI:** 10.3390/molecules23061420

**Published:** 2018-06-12

**Authors:** Mohamed El-Naggar, Wagdy M. Eldehna, Hadia Almahli, Amr Elgez, Mohamed Fares, Mahmoud M. Elaasser, Hatem A. Abdel-Aziz

**Affiliations:** 1 Chemistry Department, Faculty of Sciences, University of Sharjah, Sharjah 27272, UAE; m5elnaggar@yahoo.com; 2 Department of Pharmaceutical Chemistry, Faculty of Pharmacy, Kafrelsheikh University, Kafrelsheikh 33516, Egypt; 3 Chemistry Research Laboratory, Department of Chemistry, University of Oxford, 12 Mansfield Road, OX1 3TA Oxford, UK; hadia.almahli@yahoo.com; 4 Department of Pharmaceutical Chemistry, Faculty of Pharmacy, Egyptian Russian University, Badr City, Cairo 11829, Egypt; ph.fares@yahoo.com; 5 Department of Pharmacology and Toxicology, Faculty of Pharmacy, Egyptian Russian University, Badr City, Cairo 11829, Egypt; amro_elgez@yahoo.com; 6 School of Chemistry, University of Wollongong, Wollongong 2522, New South Wales, Australia; 7 The Regional Center for Mycology and Biotechnology, Al-Azhar University, Cairo 11759, Egypt; mmelaasser@hotmail.com; 8 Department of Applied Organic Chemistry, National Research Center, Dokki, Cairo 12622, Egypt; hatem_741@yahoo.com

**Keywords:** triple-negative breast cancer, isatin-thiazolidinone hybrids, anticancer, apoptosis, QSAR

## Abstract

In connection with our research program on the development of new isatin-based anticancer candidates, herein we report the synthesis of two novel series of thiazolidinone-isatin conjugates (**4a**–**n**) and thiazolo[3,2-*a*]benzimidazolone-isatin conjugates (**7a**–**d**), and in vitro evaluation of their antiproliferative activity towards two breast cancer cell lines; triple negative MDA-MB-231, and MCF-7. Compounds **4m** and **7b** emerged as the most active congeners against MDA-MB-231 cells (IC_50_ = 7.6 ± 0.5 and 13.2 ± 1.1 µM, respectively). Compounds **4m** and **7b** were able to provoke apoptosis in MDA-MB-231 cells, evidenced by the up-regulation of Bax and down-regulation of Bcl-2, besides boosting caspase-3 levels. Hybrid **4m** induced a fourfold increase in the percentage of cells at Sub-G_1_, with concurrent arrest in G_2_-M phase by 2.5-folds. Furthermore, hybrid **4m** resulted in a sixfold increase in the percentage of annexin V-FITC positive apoptotic MDA-MB-231 cells as compared with the control. Moreover, the cytotoxic activities of the active conjugates were assessed towards two nontumorigenic cell lines (breast MCF-10A and lung WI-38) where both conjugates **4m** and **7b** displayed mean tumor selectivity index: 9.6 and 13.9, respectively. Finally, several ADME descriptors were predicted for the active conjugates via a theoretical kinetic study.

## 1. Introduction

Breast cancer has become the most frequently diagnosed malignancy among women, and the second leading cause of cancer-related deaths in women [1,2,3]. The deficiency in the maintenance of genomic integrity, excessive exposure to estrogens and advanced age are considered as the leading reasons for a high breast cancer risk [4,5]. Breast cancer is considered as a diverse group of diseases with several intrinsic tumor subtypes that have diverse treatment modalities and long-term survival probabilities. The immunohistochemical expression of the estrogen receptor (ER), progesterone receptor (PR), and human epidermal growth factor receptor-2 (HER-2) forms the platform of characterization of clinically defined breast cancer subtypes [4]. Particularly, triple-negative breast cancers (TNBCs) lacking the expression of the (ER), (PR) and (HER-2) represent the major cause of breast cancer mortality due to their metastatic potential, invasiveness and the lack of suitable molecular treatment targets [6,7,8].

Despite an early diagnosis and the diverse therapeutic regimens available for breast cancer treatment, the development of chemo-resistance, the disease relapse, and the mortality rate are still constantly on the rise [4]. This finding highlights the need for new drug leads as more effective and affordable approaches for breast cancer therapies.

**Figure 1:**
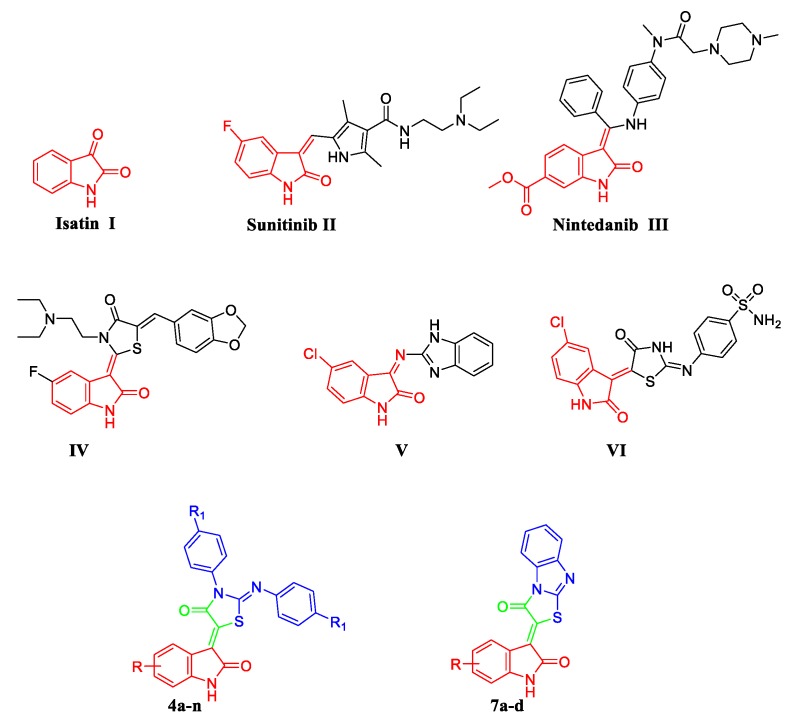
Structures of isatin-based approved anticancer drugs (**II** and **III**), some reported isatin-based hybrids with potent antiproliferative activity against breast cancer (**VII**–**IX**), and the target conjugates (**4a**–**n** and **7a**–**d**).

Pertaining to its wide presence endogenously in human and other mammalian tissues, isatin **1**, Figure 1, has emerged as a promising privileged scaffold that is endowed with diverse biological activity [9,10,11,12,13], primarily anticancer activity [14]. During the last two decades, there is a growing interest regarding development of several isatin derivatives as promising drug candidates for the treatment of different human malignancies. These research efforts have led to FDA approval of two drugs and the discovery of many isatin derivatives with diverse cellular and enzymatic targets.

Sunitinib (Sutent^®^) **II** (Figure 1), an isatin-based multitarget tyrosine kinase inhibitor, was granted its FDA approval in 2006 for the treatment of imatinib-resistant gastrointestinal stromal tumors (GIST) and advanced metastatic renal cell carcinoma (RCC) [15]. By 2014, Nintedanib (**III**, Ofev^®^, Figure 1), an orally available isatin-based triple-angiokinase inhibitor, was approved by the FDA for the management of idiopathic pulmonary fibrosis [16]. One year later, the European Medicines Agency approved Nintedanib, under a trade name Vargatef^®^, as a second-line treatment in combination with docetaxel for patients with advanced non-small cell lung cancer of adenocarcinoma who have progressed to first-line chemotherapy [17].

Over the last few years, isatin-based hybrids have attracted considerable attention as promising anticancer agents [18,19,20,21,22,23,24]. In this context, numerous research groups adopted a hybridization approach for the development of diverse isatin-thiazolidine/thiazolidinone [25,26,27,28,29] (such as compound **IV**, Figure 1) and isatin-benzimidazole (such as compound **V**, Figure 1) [30] hybrids as potent antiproliferative agents towards different breast cancer cell lines. Recently, our research group has focused on the design and synthesis of novel and potent isatin-based hybrids as promising anticancer agents [31,32,33,34,35,36,37,38,39,40]. In 2017, we developed two novel series of thiazolidinone-isatin hybrids as effective anticancer agents targeting tumor-associated carbonic anhydrase isoform IX. Among these hybrids, compound **VI** (Figure 1) displayed potent activity against MCF-7 breast cancer cell line (IC_50_ = 3.96 ± 0.21 μM) with induction of the intrinsic apoptotic mitochondrial pathway in MCF-7 cells [41].

Taking the above into account, herein we adopted the hybrid pharmacophore approach to design and synthesize two novel series of thiazolidinone-isatin (**4a**–**n**) and thiazolo[3,2-*a*]benzimidazolone-isatin conjugates (**7a**–**d**) (Figure 1), with the goal of developing potent antiproliferative agents toward TNBC MDA-MB-231, and MCF-7 breast cancer cell lines. Furthermore, the most potent antiproliferative congeners were evaluated for their apoptosis induction potential in MDA-MB-231 cells, to gain insight into the mechanism of the anticancer activity for the prepared hybrids. Finally, the cytotoxic activity of the active conjugates was tested towards nontumorigenic human normal lung fibroblast cell line (WI-38) to investigate the safety of the newly prepared conjugates.

## 2. Results

### 2.1. Chemistry

**Scheme 1:**
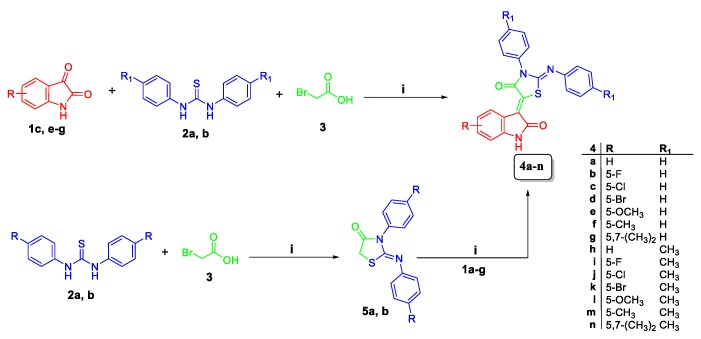
Synthesis of target hybrids **4a**–**n**; reagents and conditions: (**i**) glacial acetic acid/sodium acetate/reflux 3–4 h.

**Scheme 2:**
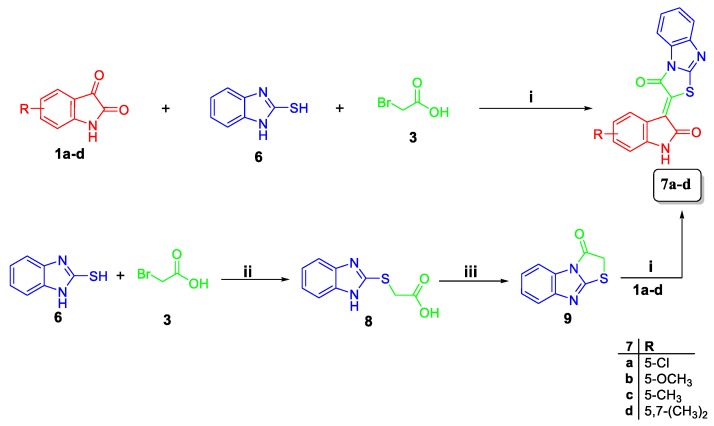
Synthesis of target hybrids **7a**–**d**; Reagents and conditions: (**i**) glacial acetic acid/sodium acetate/reflux 2 h; (**ii**) ethanol/ KOH/reflux 1 h; (**iii**) acetic anhydride/pyridine 100 °C 1 h.

The target conjugates **4a**–**n** and **7a**–**d** were synthesized adopting the chemical pathways outlined in Scheme 1 and Scheme 2. In Scheme 1, preparation of conjugates **4a**–**n** was accomplished via two synthetic routes. The first route comprised a one-pot three-component synthesis, involving a Knoevenagel condensation, followed by cyclization among isatins **1a**–**g**, utilising an equimolar amount of *N*,*N*′-diphenyl/*p*-tolyl thiourea **2a**,**b** and bromoacetic acid **3** in refluxing glacial acetic acid in the presence of sodium acetate to furnish conjugates **4a**–**n**. This route proved successful to prepare conjugates **4a**–**n** in high yields; 70–86% (Scheme 1). Alternatively, *N*,*N*′-diphenyl/*p*-tolyl thiourea **2a**,**b** reacted with bromoacetic acid **3** to furnish compounds **5a**,**b**, which subsequently condensed with isatins **1a**–**g** in acetic acid in the presence of sodium acetate to afford the target conjugates **4a**–**n**, with overall 55–68% yield over the two steps (Scheme 1).

Similar to Scheme 1, target conjugates **7a**–**d** were prepared in a one-pot three-component reaction of isatins **1c**, **e**–**g** with equimolar amounts of 2-mercaptobenzimidazole **6** and bromoacetic acid **3** in refluxing glacial acetic acid in the presence of sodium acetate to produce conjugates **7a**–**d**, in good yields; 80–88% (Scheme 2). The latter conjugates prepared through cyclization of 2-((benzimidazol-2-yl)thio)acetic acid **8** into compound **9**, with subsequent condensation with isatins **1c**, **e**–**g**, with low overall yield; 45–61%.

Postulated structures of the newly synthesized conjugates **4a**–**n** and **7a**–**d** were in full agreement with their spectral and elemental analyses data.

### 2.2. Biological Evaluation

#### 2.2.1. In Vitro Antiproliferative Activity

The in vitro antiproliferative activity of the newly synthesized conjugates **4a**–**n** and **7a**–**d** was evaluated against breast cancer MDA-MB-231 and MCF-7 cell lines, following the Crystal Violet (CV) cell cytotoxicity assay [41]. Doxorubicin was included in this assay as a positive control, and Sunitinib was used as a reference drug. The results were expressed as median growth inhibitory concentration (IC_50_) values that represent the compounds concentrations required to afford a 50% inhibition of cell growth after 48 h of incubation, compared to untreated controls (Table 1).

Breast cancer is composed of multiple subtypes with distinct morphologies and clinical implications. In this study, the sensitivity of two different breast cancer subtypes towards the target conjugates was tested to examine if the target compounds possess selective growth inhibitory activity toward certain subtype. MCF-7 is a Luminal A breast cancer cells which is hormone-receptor positive (estrogen-receptor and/or progesterone-receptor positive), HER2 negative, and has low levels of the protein Ki-67, which helps control how fast cancer cells grow. Luminal A cancers are low-grade, tend to grow slowly and have the best prognosis. Whereas, MDA-MB-231 is a triple-negative/basal-like breast cancer cell line, which is hormone-receptor negative (estrogen-receptor and progesterone-receptor negative) and HER2 negative. This type of cancer is more common in women with BRCA1 gene mutations.

From the obtained results, it was obvious that many of the target conjugates **4a**–**n** and **7a**–**d** have excellent to modest growth inhibitory activity against the tested breast cancer cell lines; MDA-MB-231 and MCF-7. Also, it was noted that MDA-MB-231 cells is more sensitive to the influence of the target conjugates than MCF-7 cells, especially for the thiazolobenzimidazole conjugates **7a**–**d**.

Concerning activity against MDA-MB-231 cells, conjugate **4m** was the most active one and showed potent antiproliferative activity with IC_50_ 7.6 ± 0.5 μM, in comparison to the standard drug doxorubicin (IC_50_ = 4.7 ± 0.4 μM). Furthermore, compounds **4c**, **4d**, **7b** and **7d** displayed good activity against MDA-MB-231 cells with IC_50_ values of 24.1 ± 2.1, 15.7 ± 0.9, 13.2 ± 1.1 and 23.5 ± 1.0 μM, respectively. Moreover, conjugates **4e**, **4g**, **4l**, **4n**, **7a** and **7c** possessed moderate activity with an IC_50_ range 36.3 ± 2.9–47.1 ± 4.1 µM.

On the other hand, examination of the antiproliferative activity in MCF-7 cells elucidated that conjugate **4m** had the best growth inhibitory activity (IC_50_ = 8.4 ± 0.5), with 2.2-fold decreased activity than doxorubicin (IC_50_ = 3.80 ± 0.4 μM). Furthermore, hybrid **4g** showed good antiproliferative activity against MCF-7 cancer cell line (IC_50_ = 17.1 ± 1.1). Also, hybrids **4c**, **4f**, **4n**, **7b** and **7d** were moderately active towards MCF-7 cells with IC_50_ values of 29.2 ± 2.5, 22.8 ± 2.0, 23.7 ± 1.9, 21.7 ± 1.5 and 27.6 ± 2.4, respectively.

It is worth highlighting that both target thiazolidinone-isatin conjugates (**4a**–**n**) and thiazolo[3,2-*a*]benzimidazolone-isatin conjugates (**7a**–**d**), didn’t display significant selectivity in their growth inhibitory activities towards the tested cell line; TNBC MDA-MB-231 or MCF-7.

#### 2.2.2. In Vitro Cytotoxic Activity towards Nontumorigenic Human WI-38 and MCF-10A Cells

The cytotoxic activity of the active conjugates towards MDA-MB-231 cells (**4c**–**e**, **4g**, **4l**–**n** and **7a**–**d**) was examined against nontumorigenic human lung fibroblast cell line (WI-38) and human breast epithelial cell line (MCF-10A) to investigate the potential safety of the newly prepared conjugates towards the normal cells. Cultures derived from human fibrocystic mammary tissue (MCF-10A) are nontumorigenic and possess the features of primary cultures of breast tissue including dome formation [42]. The results were expressed as IC_50_ values, and selectivity index was calculated (Table 2). Most of the tested hybrids (**4c**–**e**, **4g**, **4l**–**n** and **7a**–**d**) exhibited non-significant cytotoxic impact towards WI-38 and MCF-10A cells with IC_50_ range 49.1 ± 1.9–353.0 ± 17.1 and 73.1 ± 2.7–250.3 ± 9.8 μM, respectively. Compounds **4m** and **7a**–**d** displayed good selectivity index range 6.0–13.9, thereby providing a good safety profile as anticancer agents.

#### 2.2.3. Apoptosis Induction in TNBC MDA-MB-231 Cells

In the current medical era, induction of apoptosis in cancer cells has emerged as one of the most successful strategies for the development of cancer therapies [43,44,45]. As mentioned before, **4m** and **7b** emerged as the most active hybrids towards MDA-MB-231 cells. Consequently, we examined the ability of conjugates **4m** and **7b** to provoke apoptosis in MDA-MB-231 cells to determine the principle mechanism for their antiproliferative activity.

##### Effects on Mitochondrial Apoptosis Pathway Proteins Bcl-2 and Bax

Several members of the human Bcl-2 family of apoptosis-regulating proteins have been discovered, including antiapoptotic proteins such as Bcl-2 protein, and structurally similar pro-apoptotic proteins such as Bax protein, the first anti-death gene identified [44,45,46]. Bcl-2-family proteins regulate all major types of cell death, including apoptosis, necrosis and autophagy, therefore operating as nodal points at the convergence of diverse pathways with broad relevance to oncology.

**Figure 2:**
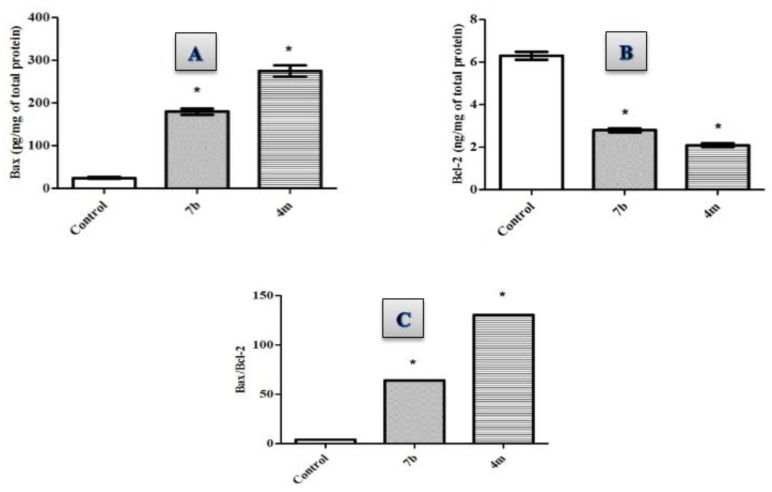
Effect of compounds **4m** and **7b** on the protein levels of (**A**) Bax; (**B**) Bcl-2; (**C**) Bax/Bcl2 ratio in MDA-MB-231 cells treated with the compounds at their IC_50_ concentrations against control (1% DMSO). Data are mean ±S.D. (*n* = 3). The experiment was done in triplicates. * Significantly different from control at *p* < 0.05.

In our study, we investigated the effect of conjugates **4m** and **7b** towards the level of the antiapoptotic Bcl2 and the level of the pro-apoptotic Bax, (Table 3, Figure 2). As displayed in Table 2, hybrid **4m** induced the protein expression of Bax with 11.1 folds of the control while 7.3 folds were evaluated for hybrid **7b**. On the other hand, treatment of MDA-MB-231 cancer cells with conjugates **4m** and **7b** significantly downregulated the expression levels of the antiapoptotic protein Bcl-2 by 66.6 and 55.1%, respectively, compared to the control.

The ratio between Bax (apoptosis inducer) and Bcl-2 (apoptosis suppressor) is an important parameter and gave more profound insight into the apoptotic activity of the compounds as a determining factor for cell fate regulation, and a key indicator of therapeutic response to chemotherapy [47]. Calculating the values for Bax/Bcl2 ratio indicated that conjugates **4m** and **7b** boosted the Bax/Bcl-2 ratio by 33- and 16-folds, respectively, compared to the control. Conclusively, the ability of conjugates **4m** and **7b** to up-regulate Bax level, down-regulate Bcl2 level while significantly boosting the Bax/Bcl2 ratio proves their effectiveness as apoptosis inducers.

##### Effects on the Levels of Active Caspase-3 (Key Executor of Apoptosis)

**Figure 3:**
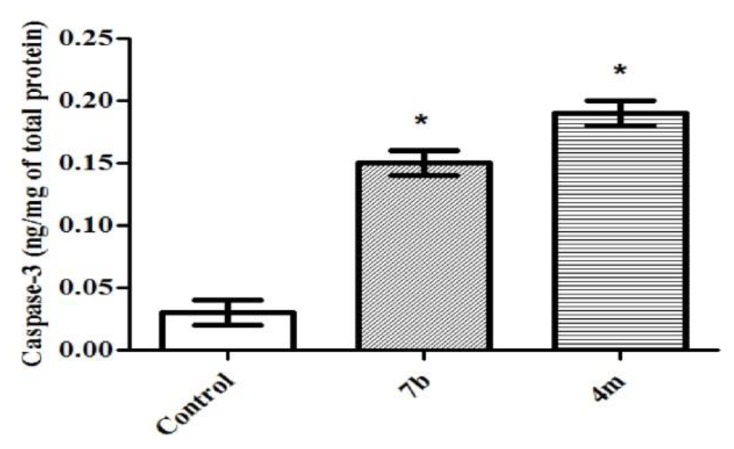
Effect of compounds **4m** and **7b** on the protein levels of caspase-3 in MDA-MB-231 cells treated with the compounds at their IC_50_ concentrations against control (1% DMSO). Data are mean ±S.D. (*n* = 3). The experiments were done in triplicate. * Significantly different from control at *p* < 0.05.

The down-regulation of the antiapoptotic Bcl-2 leads to an increase in the levels of free pro-apoptotic Bax which in turn accumulates at the inner mitochondrial membrane forming channels, altering membrane permeability. Accordingly, the apoptotic factors can leak into the cytoplasm resulting in the caspases cascade activation. As a key executioner protease, caspase-3 is activated by upstream initiator caspases as caspase-9 [48]. Thence, the elevated Bax/Bcl-2 ratios obtained herein triggered the examination of the protein expression levels of active caspases-3. Treatment of TNBC MDA-MB-231 cells with conjugates **4m** and **7b** led to a significant increase in the active caspase-3 levels by 6.4 and 4.9 folds, respectively, compared to control (Table 3, Figure 3).

##### Cell Cycle Analysis

**Figure 4:**
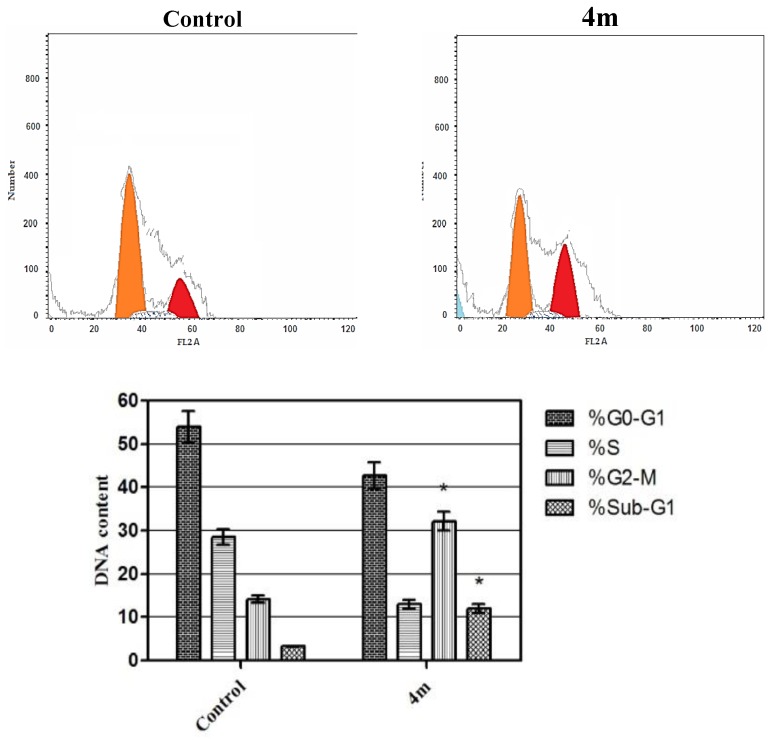
Effect of compound **4m** on the phases of cell cycle of MDA-MB-231 cells. * Significantly different from control at *p* < 0.05. (Two-way ANOVA test).

The effect of hybrid **4m** on cell cycle progression was evaluated in TNBC MDA-MB-231 cells after 24 h of treatment (Figure 4). Such impact was illustrated by DNA flow cytometric assay where MDA-MB-231 cells were treated with conjugate **4m** at its IC_50_ concentration.

As displayed in Figure 4, exposure of MDA-MB-231 cells to hybrid **4m** resulted in a significant rise in the percentage of cells at Sub-G_1_ by 4-folds, with concurrent significant arrest in the G_2_-M phase by 2.5-folds compared to control. Arrest of G_2_-M phase and alteration of the Sub-G_1_ phase were significant remarks for hybrid **4m** to prompt apoptosis in TNBC MDA-MB-231 cells.

##### Annexin V-FITC Apoptosis Assay

**Figure 5:**
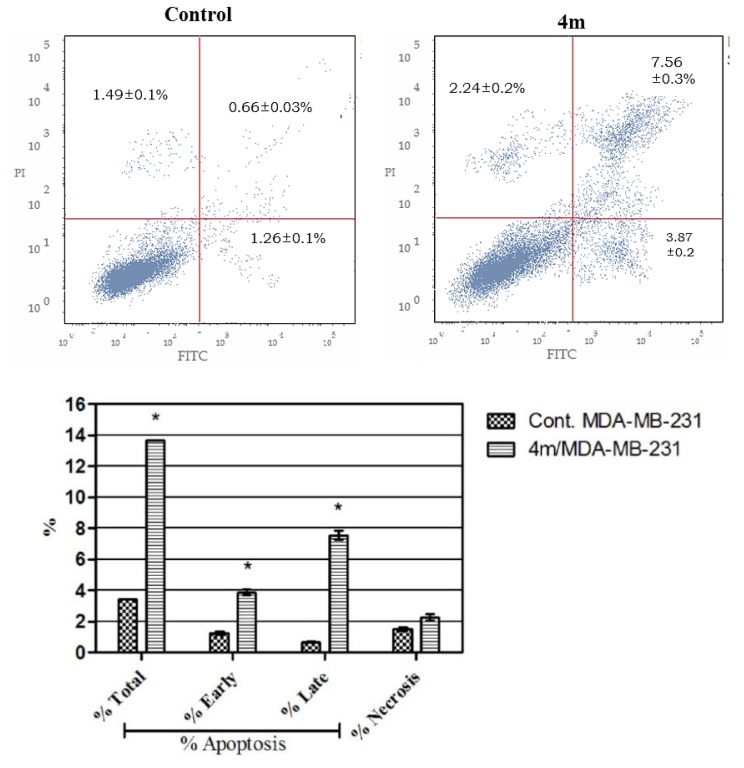
Effect of compound **4m** on the percentage of annexin V-FITC-positive staining in MDA-MB-231 cells. The experiments were done in triplicates. The four quadrants identified as: **LL**, viable; **LR**, early apoptotic; **UR**, late apoptotic; **UL**, necrotic. * Significantly different from control at *p* < 0.05. (One-way ANOVA test).

Annexin V-based flow cytometry assay elucidates either that cell death is achieved via programmed apoptosis or nonspecific necrosis [49]. The apoptotic impact of conjugate **4m** was further proved by Annexin V-FITC/PI (AV/PI) dual staining analysis to examine the occurrence of phosphatidylserine externalization (Figure 5). Flow cytometric analysis revealed that treatment of TNBC MDA-MB-231 cells with conjugate **4m** resulted in a significant elevation in the percent of annexin V-FITC-positive apoptotic cells, including both the early and late apoptotic phases (UR + LR), from 1.92% to 11.43% which represents about 6-folds increase as compared with the control.

In conclusion, enhanced expression of the pro-apoptotic protein Bax in addition to the reduced expression of the antiapoptotic protein Bcl-2, as well as the up-regulated active caspase-3 levels together with a harmonized increase in the Bax/Bcl-2 ratio, suggests that the antiproliferative effect of the target conjugates might be attributed, at least in part, to the induction of the intrinsic apoptotic mitochondrial pathway.

Collectively, these data have highlighted conjugate **4m** as an ideal lead compound for further optimization and development as an effective anti-breast cancer therapy.

### 2.3. In Silico ADME Profiling

In order to examine drug-like physicochemical and pharmacokinetics properties of the target conjugates, certain ADME descriptors for the prepared conjugates (**4a**–**n** and **7a**–**d**) were assessed through a computer-aided theoretical kinetic study using Discovery Studio software (Accelrys, San Diego, CA, USA), [50,51] Table 4.

The tested conjugates exhibited low aqueous solubility levels, except compounds **7b** and **7c**, which showed better solubility levels. The tested conjugates displayed good levels of human intestinal absorption, except compounds **4i**–**k**, **4m** and **4n**, which showed moderate intestinal absorption levels. On the other hand, the investigated conjugates were predicted to be CYP2D6 non-inhibitors, Table 4.

## 3. Conclusions

In summary, herein we report the synthesis of two novel series of thiazolidinone-isatin conjugates (**4a**–**n**) and thiazolo[3,2-*a*]benzimidazolone-isatin conjugates (**7a**–**d**). All the prepared conjugates were evaluated for their antiproliferative activity towards breast cancer cell lines MDA-MB-231, and MCF-7. Conjugates **4m** and **7b** were the most active members towards MDA-MB-231 (IC_50_ = 7.6 ± 0.51 and 13.2 ± 1.07 µM, respectively). Conjugates **4d** and **4m** emerged as the most active derivatives against MCF-7 cells with IC_50_ values of 17.1 ± 1.14 and 8.4 ± 0.47 μM, respectively. Conjugates **4m** and **7b** induced apoptosis in MDA-MB-231 cells, confirmed by the up-regulation of the Bax, downregulation of the Bcl-2 and boosting caspase-3 levels. Compound **4m** arrested the G_2_-M phase by 2.5-folds with concurrent significant increase in the percentage of cells at Sub-G_1_ by 4-folds, compared to control. Also, **4m** showed significant increase in the percentage of annexin V-FITC positive apoptotic cells from 1.92% to 11.43%, approximately a 6-folds increase as compared with the control. Finally, the cytotoxic activity of the active conjugates (**4c**–**e**, **4g**, **4l**–**n** and **7a**–**d**) were tested towards nontumorigenic human lung fibroblast WI-38 cell line and breast MCF-10A cell line, and compounds **4m** and **7a**–**d** displayed good selectivity index range 6.0–13.9, thereby providing a good safety profile as anticancer agents.

## 4. Experimental

### 4.1. Chemistry

#### 4.1.1. General 

Using a Stuart melting point apparatus, melting points were measured and uncorrected. Using Schimadzu FT-IR 8400S spectrophotometer (Shimadzu, Kyoto, Japan), Infrared (IR) Spectra were recorded as KBr disks. NMR Spectra were recorded on a Bruker spectrophotometer (Bruker, Karlsruhe, Germany). ^1^H spectrum was run at 400 MHz and ^13^C spectrum was run at 100 MHz in deuterated dimethylsulfoxide (DMSO-*d_6_*). All coupling constant (*J*) values are given in hertz. The abbreviations used are as follows: *s*, singlet; *d*, doublet; *m*, multiplet. Elemental analyses were done at the Regional Center for Microbiology and Biotechnology, Al-Azhar University, Egypt. Mass spectral data were given by a GCMS-QP1000 EX spectrometer (Shimadzu, Kyoto, Japan) at 70 e.V. High-resolution mass spectra were obtained using a Bruker MicroTOF spectrometer (Bruker Daltonics, Bremen, Germany). Compounds **5a**,**b** [52], **8** [53], and **9** [54] were previously prepared.

#### 4.1.2. General Procedure for the Preparation of Target Compounds **4a**–**n**

Route A:

A mixture of isatins **1a**–**g** (1 mmoL) with an equimolar amount of *N*,*N*′-diphenyl/(*p*-tolyl)thiourea **2a**,**b** (1 mmoL) and bromoacetic acid **3** (0.14 g, 1 mmoL) in glacial acetic acid (10 mL) in the presence of sodium acetate (0.16 gm, 2 mmoL), was heated under reflux for 3 h. The formed solid was filtered off while hot, washed with hot ethanol, dried and recrystallized from DMF to furnish the target hybrids **4a**–**n**.

Route B:

To a hot solution of *N*,*N*′-diphenyl/(*p*-*tolyl*)thiourea **2a**,**b** (1 mmoL) and sodium acetate (0.16 gm, 2 mmoL) in glacial acetic acid (10 mL), bromoacetic acid **3** (0.14 gm, 1 mmoL) was added. The reaction mixture was refluxed for 4 h then allowed to cool to room temperature. The obtained solid was filtered off, washed with water and dried to give intermediates **3a**,**b**. The later intermediates were added, without further purification, to a stirred solution of the appropriate isatin derivative **1a**–**g** (1 mmoL) and sodium acetate (0.16 gm, 2 mmoL) in glacial acetic acid (5 mL) then the reaction mixture was refluxed for 3 h. The formed solid was filtered off, washed with ethanol and recrystallized from DMF to give compounds **4a**–**n**.

5-(2-Oxoindolin-3-ylidene)-3-phenyl-2-(phenylimino)thiazolidin-4-one (**4a**)

Red powder (yield 83%), m.p. > 300 °C; IR (KBr, *ν* cm^−1^): 3251 (NH) and 1687 (C=O); ^1^H-NMR (DMSO-*d*_6_, 400 MHz) *δ ppm*: 6.95 (d, *J* = 7.7 Hz, 1H, Ar-H), 6.99 (d, *J* = 7.3 Hz, 2H, Ar-H), 7.04 (t, *J* = 7.3 Hz, 1H, Ar-H), 7.17 (t, *J* = 7.4 Hz, 1H, Ar-H), 7.35–7.43 (m, 3H, Ar-H), 7.49–7.54 (m, 1H, Ar-H), 7.58 (d, *J* = 7.7 Hz, 4H, Ar-H), 8.77 (d, *J* = 7.7 Hz, 1H, Ar-H), 11.18 (s, 1H, NH isatin, D_2_O exchangeable); MS, *m/z* [%]: 397.0 [M^+^, 100]; Anal. Calcd. for C_23_H_15_N_3_O_2_S: C, 69.51; H, 3.80; N, 10.57; found C, 69.34; H, 3.91; N, 10.83; HRMS *m*/*z* 398.09583 M^+^ + 1, calcd for C_23_H_15_N_3_O_2_S: 398.09577.

5-(5-Fluoro-2-oxoindolin-3-ylidene)-3-phenyl-2-(phenylimino)thiazolidin-4-one (**4b**)

Orange powder (yield 75%), m.p. > 300 °C; IR (KBr, *ν* cm^−1^): 3343 (NH) and 1683 (C=O); ^1^H-NMR (DMSO-*d*_6_, 400 MHz) *δ ppm*: 6.81–6.87 (m, 3H, Ar-H), 7.22 (d, *J* = 7.8 Hz, 2H, Ar-H), 7.42–7.60 (m, 7H, Ar-H), 8.57 (d, *J* = 7.2 Hz, 1H, Ar-H), 11.25 (s, 1H, NH isatin, D_2_O exchangeable); Anal. Calcd. for C_23_H_14_FN_3_O_2_S: C, 66.50; H, 3.40; N, 10.11; found C, 66.78; H, 3.26; N, 10.40.

5-(5-Chloro-2-oxoindolin-3-ylidene)-3-phenyl-2-(phenylimino)thiazolidin-4-one (**4c**)

Light brown powder (yield 80%), m.p. > 300 °C; IR (KBr, *ν* cm^−1^): 3257 (NH) and 1687 (C=O); ^1^H-NMR (DMSO-*d*_6_, 400 MHz) *δ ppm*: 6.89 (d, *J* = 8.4 Hz, 1H, Ar-H), 6.97 (d, *J* = 7.6 Hz, 2H, Ar-H), 7.24–7.33 (m, 3H, Ar-H), 7.51–7.63 (m, 6H, Ar-H), 8.71 (d, *J* = 2.0 Hz, 1H, Ar-H), 11.29 (s, 1H, NH isatin, D_2_O exchangeable); Anal. Calcd. for C_23_H_14_ClN_3_O_2_S: C, 63.96; H, 3.27; N, 9.73; found C, 64.23; H, 3.41; N, 10.01.

5-(5-Bromo-2-oxoindolin-3-ylidene)-3-phenyl-2-(phenylimino)thiazolidin-4-one (**4d**)

Red powder (yield 85%), m.p. > 300 °C; IR (KBr, *ν* cm^−1^): 3308 (NH) and 1689 (C=O); ^1^H-NMR (DMSO-*d*_6_, 400 MHz) *δ ppm*: 6.93 (d, *J* = 8.3 Hz, 1H, Ar-H), 6.99 (d, *J* = 7.3 Hz, 2H, Ar-H), 7.20 (t, *J* = 7.4 Hz, 1H, Ar-H), 7.42 (t, *J* = 7.8 Hz, 2H, Ar-H), 7.65–7.49 (m, 6H, Ar-H), 8.96 (d, *J* = 2.0 Hz, 1H, Ar-H), 11.37 (s, 1H, NH isatin, D_2_O exchangeable); Anal. Calcd. for C_23_H_14_BrN_3_O_2_S: C, 57.99; H, 2.96; N, 8.82; found C, C, 58.17; H, 3.08; N, 9.04; HRMS *m*/*z* 476.00635 M^+^ + 1, calcd for C_23_H_14_BrN_3_O_2_S: 476.00629.

5-(5-Methoxy-2-oxoindolin-3-ylidene)-3-phenyl-2-(phenylimino)thiazolidin-4-one (**4e**)

Red powder (yield 70%), m.p. > 300 °C; IR (KBr, *ν* cm^−1^): 3340 (NH) and 1692 (C=O); ^1^H-NMR (DMSO-*d*_6_, 400 MHz) *δ ppm*: 3.72 (s, 3H, -OCH_3_), 6.86 (d, *J* = 8.5 Hz, 1H, Ar-H), 7.05–6.94 (m, 3H, Ar-H), 7.19 (t, *J* = 7.4 Hz, 1H, Ar-H), 7.41 (t, *J* = 7.8 Hz, 2H, Ar-H), 7.57–7.46 (m, 1H, Ar-H), 7.59 (d, *J* = 4.3 Hz, 4H, Ar-H), 8.48 (d, *J* = 2.6 Hz, 1H, Ar-H), 11.02 (s, 1H, NH isatin, D_2_O exchangeable); ^13^C-NMR (DMSO-*d*_6_, 100 MHz) *δ ppm*: 56.10, 111.14, 113.90, 118.24, 121.09, 121.31 (2C), 125.03, 125.50, 129.18 (2C), 129.56 (2C), 129.85 (2C), 132.14, 135.05, 137.54, 137.57, 147.76, 153.77, 154.83, 166.10, 169.05; MS, *m/z* [%]: 427 [M^+^, 100]; Anal. Calcd. for C_24_H_17_N_3_O_3_S: C, 67.43; H, 4.01; N, 9.83; found C, 67.70; H, 4.19; N, 10.02; HRMS *m*/*z* 428.10632 M^+^ + 1, calcd for C_24_H_17_N_3_O_3_S: 428.10634.

5-(5-Methyl-2-oxoindolin-3-ylidene)-3-phenyl-2-(phenylimino)thiazolidin-4-one (**4f**)

Red powder (yield 75%), m.p. > 300 °C; IR (KBr, *ν* cm^−1^): 3285 (NH) and 1684 (C=O); ^1^H-NMR (DMSO-*d*_6_, 400 MHz) *δ ppm*: 2.28 (s, 3H, -CH_3_), 6.83 (d, *J* = 8.0 Hz, 1H, Ar-H), 6.98 (d, *J* = 7.2 Hz, 2H, Ar-H), 7.18 (d, *J* = 7.2 Hz, 2H, Ar-H), 7.39 (t, *J* = 7.6 Hz, 2H, Ar-H), 7.52–7.59 (m, 5H, Ar-H), 8.64 (s, 1H, Ar-H), 11.10 (s, 1H, NH isatin, D_2_O exchangeable); MS, *m/z* [%]: 411 [M^+^, 100]; Anal. Calcd. for C_24_H_17_N_3_O_2_S: C, 70.06; H, 4.16; N, 10.21; found C, 69.84; H, 4.38; N, 10.38.

5-(5,7-Dimethyl-2-oxoindolin-3-ylidene)-3-phenyl-2-(phenylimino)thiazolidin-4-one (**4g**)

Brown powder (yield 73%), m.p. > 300 °C; IR (KBr, *ν* cm^−1^): 3328 (NH) and 1682 (C=O); ^1^H-NMR (DMSO-*d*_6_, 400 MHz) *δ ppm*: 2.17 (s, 3H, -CH_3_), 2.23 (s, 3H, -CH_3_), 6.84 (s, 1H, Ar-H), 6.97 (d, *J* = 7.6 Hz, 2H, Ar-H), 7.19 (d, *J* = 7.6 Hz, 2H, Ar-H), 7.41 (t, *J* = 7.6 Hz, 2H, Ar-H), 7.49–7.58 (m, 4H, Ar-H), 8.43 (s, 1H, Ar-H), 11.07 (s, 1H, NH isatin, D_2_O exchangeable); MS, *m/z* [%]: 425 [M^+^, 100]; Anal. Calcd. for C_25_H_19_N_3_O_2_S: C, 70.57; H, 4.50; N, 9.88; found C, 70.79; H, 4.61; N, 10.15.

5-(2-Oxoindolin-3-ylidene)-3-(*p*-tolyl)-2-(*p*-tolylimino)thiazolidin-4-one (**4h**)

Red powder (yield 70%), m.p. > 300 °C; IR (KBr, *ν* cm^−1^): 3271 (NH) and 1687 (C=O); ^1^H-NMR (DMSO-*d*_6_, 400 MHz) *δ ppm*: 2.33 (s, 3H, -CH_3_), 2.41 (s, 3H, -CH_3_), 6.93 (d, *J* = 7.6 Hz, 1H, Ar-H), 6.98 (d, *J* = 7.2 Hz, 2H, Ar-H), 7.06 (t, *J* = 7.3 Hz, 1H, Ar-H), 7.21 (d, *J* = 8.0 Hz, 2H, Ar-H), 7.32–76 (m, 3H, Ar-H), 7.42 (d, *J* = 8.0 Hz, 2H, Ar-H), 8.75 (d, *J* = 7.6 Hz, 1H, Ar-H), 11.27 (s, 1H, NH isatin, D_2_O exchangeable); Anal. Calcd. for C_25_H_19_N_3_O_2_S: C, 70.57; H, 4.50; N, 9.88; found C, 70.64; H, 4.64; N, 10.06.

5-(5-Fluoro-2-oxoindolin-3-ylidene)-3-(*p*-tolyl)-2-(*p*-tolylimino)thiazolidin-4-one (**4i**)

Orange powder (yield 75%), m.p. > 300 °C; IR (KBr, *ν* cm^−1^): 3290 (NH) and 1686 (C=O); ^1^H-NMR (DMSO-*d*_6_, 400 MHz) *δ ppm*: 2.32 (s, 3H, -CH_3_), 2.41 (s, 3H, -CH_3_), 6.87 (d, *J* = 8.2 Hz, 2H, Ar-H), 6.92–6.95 (m, 1H, Ar-H), 7.26–7.15 (m, 3H, Ar-H), 7.38 (d, *J* = 8.3 Hz, 2H, Ar-H), 7.44 (d, *J* = 8.3 Hz, 2H, Ar-H), 8.57 (dd, *J* = 10.4, 2.7 Hz, 1H, Ar-H), 11.23 (s, 1H, NH isatin, D_2_O exchangeable); ^13^C-NMR (DMSO-*d*_6_, 100 MHz) *δ ppm*: 31.16 (2C), 118.67, 121.16 (2C), 121.30, 124.30, 128.77 (2C), 130.00 (2C), 130.33 (2C), 131.84, 132.32, 134.11, 134.28, 138.76, 140.06, 145.54, 153.37, 155.40, 156.73, 165.92, 168.63; MS, *m/z* [%]: 443 [M^+^, 100]; Anal. Calcd. for C_25_H_18_FN_3_O_2_S: C, 67.71; H, 4.09; N, 9.47; found C, 68.03; H, 4.35; N, 9.29; HRMS *m*/*z* 444.11771 M^+^ + 1, calcd for C_25_H_18_FN_3_O_2_S: 444.11765.

5-(5-Chloro-2-oxoindolin-3-ylidene)-3-(*p*-tolyl)-2-(*p*-tolylimino)thiazolidin-4-one (**4j**)

Orange powder (yield 73%), m.p. > 300 °C; IR (KBr, *ν* cm^−1^): 3223 (NH) and 1691 (C=O); ^1^H-NMR (DMSO-*d*_6_, 400 MHz) *δ ppm*: 2.32 (s, 3H, -CH_3_), 2.41 (s, 3H, -CH_3_), 6.87 (d, *J* = 8.2 Hz, 2H, Ar-H), 6.96 (d, *J* = 8.4 Hz, 1H, Ar-H), 7.21 (d, *J* = 8.1 Hz, 2H, Ar-H), 7.50–7.35 (m, 5H, Ar-H), 8.82 (d, *J* = 2.2 Hz, 1H, Ar-H), 11.34 (s, 1H, NH isatin, D_2_O exchangeable); Anal. Calcd. for C_25_H_18_ClN_3_O_2_S: C, 65.28; H, 3.94; N, 9.14; found C, 65.41; H, 4.12; N, 9.32; HRMS *m*/*z* 460.08807 M^+^ + 1, Calcd. for C_25_H_18_ClN_3_O_2_S: 460.08810.

5-(5-Bromo-2-oxoindolin-3-ylidene)-3-(*p*-tolyl)-2-(*p*-tolylimino)thiazolidin-4-one (**4k**)

Red powder (yield 86%), m.p. > 300 °C; IR (KBr, *ν* cm^−1^): 3296 (NH) and 1690 (C=O); ^1^H-NMR (DMSO-*d*_6_, 400 MHz) *δ ppm*: 2.33 (s, 3H, -CH_3_), 2.43 (s, 3H, -CH_3_), 6.91 (d, *J* = 8.0 Hz, 2H, Ar-H), 7.01 (d, *J* = 8.0 Hz, 1H, Ar-H), 7.25 (d, *J* = 7.6 Hz, 2H, Ar-H), 7.39–7.51 (m, 5H, Ar-H), 9.03 (s, 1H, Ar-H), 11.41 (s, 1H, NH isatin, D_2_O exchangeable); Anal. Calcd. for C_25_H_18_BrN_3_O_2_S: C, 59.53; H, 3.60; N, 8.33; found C, 59.80; H, 3.78; N, 8.59.

5-(5-Methoxy-2-oxoindolin-3-ylidene)-3-(*p*-tolyl)-2-(*p*-tolylimino)thiazolidin-4-one (**4l**)

Red powder (yield 75%), m.p. > 300 °C; IR (KBr, *ν* cm^−1^): 3256 (NH) and 1689 (C=O); ^1^H-NMR (DMSO-*d*_6_, 400 MHz) *δ ppm*: 2.32 (s, 3H, -CH_3_), 2.40 (s, 3H, -CH_3_), 3.71 (s, 3H, -OCH_3_), 6.83–6.88 (m, 3H, Ar-H), 6.95 (d, *J* = 7.8 Hz, 1H, Ar-H), 7.19 (d, *J* = 8.0 Hz, 2H, Ar-H), 7.37 (d, *J* = 8.0 Hz, 2H, Ar-H), 7.42 (d, *J* = 8.0 Hz, 2H, Ar-H), 8.47 (s, 1H, Ar-H), 11.01 (s, 1H, NH isatin, D_2_O exchangeable); ^13^C-NMR (DMSO-*d*_6_, 100 MHz) *δ ppm*: 20.97, 21.27, 55.99, 111.14, 113.89, 118.27, 121.18, 121.22, 125.59, 128.62, 128.86, 129.00, 130.02 (2C), 130.30 (2C), 132.35, 132.56, 134.29, 137.53, 138.92, 145.49, 153.83, 155.02, 166.06, 169.03; MS, *m/z* [%]: 455 [M^+^, 100]; Anal. Calcd. for C_26_H_21_N_3_O_3_S: C, 68.55; H, 4.65; N, 9.22; found C, 68.47; H, 4.60; N, 9.53; HRMS *m*/*z* 456.13754 M^+^ + 1, calcd for C_26_H_21_N_3_O_3_S: 456.13764.

5-(5-Methyl-2-oxoindolin-3-ylidene)-3-(*p*-tolyl)-2-(*p*-tolylimino)thiazolidin-4-one (**4m**)

Red powder (yield 80%), m.p. > 300 °C; IR (KBr, *ν* cm^−1^): 3358 (NH) and 1693 (C=O); ^1^H-NMR (DMSO-*d*_6_, 400 MHz) *δ ppm*: 2.26 (s, 3H, -CH_3_), 2.32 (s, 3H, -CH_3_), 2.41 (s, 3H, -CH_3_), 6.82 (d, *J* = 8.0 Hz, 1H, Ar-H), 6.91 (d, *J* = 7.2 Hz, 2H, Ar-H), 7.19–7.23 (m, 3H, Ar-H), 7.35 (d, *J* = 7.6 Hz, 2H, Ar-H), 7.43 (d, *J* = 7.6 Hz, 2H, Ar-H), 8.54 (s, 1H, Ar-H), 11.19 (s, 1H, NH isatin, D_2_O exchangeable); MS, *m/z* [%]: 439 [M^+^, 100]; Anal. Calcd. for C_26_H_21_N_3_O_2_S: C, 71.05; H, 4.82; N, 9.56; found C, 70.89; H, 4.96; N, 9.74.

5-(5,7-Dimethyl-2-oxoindolin-3-ylidene)-3-(*p*-tolyl)-2-(*p*-tolylimino)thiazolidin-4-one (**4n**)

Brown powder (yield 71%), m.p. > 300 °C; IR (KBr, *ν* cm^−1^): 3319 (NH) and 1685 (C=O); ^1^H-NMR (DMSO-*d*_6_, 400 MHz) *δ ppm*: 2.19 (s, 3H, -CH_3_), 2.24 (s, 3H, -CH_3_), 2.32 (s, 3H, -CH_3_), 2.41 (s, 3H, -CH_3_), 6.87 (d, *J* = 8.2 Hz, 2H, Ar-H), 7.02 (s, 1H, Ar-H), 7.21 (d, *J* = 8.1 Hz, 2H, Ar-H), 7.37 (d, *J* = 8.3 Hz, 2H, Ar-H), 7.43 (d, *J* = 8.3 Hz, 2H, Ar-H), 8.49 (s, 1H, Ar-H), 11.11 (s, 1H, NH isatin, D_2_O exchangeable); ^13^C-NMR (DMSO-*d*_6_, 100 MHz) *δ ppm*: 18.90, 21.35, 28.18 (2C), 119.54, 120.90 (2C), 126.31, 128.86 (2C), 129.87 (2C), 130.30 (2C), 130.93, 131.46, 132.59, 134.14, 138.97, 139.89, 143.02, 145.42, 153.76, 157.33, 163.30, 165.80, 169.33; MS, *m/z* [%]: 453 [M^+^, 100]; Anal. Calcd. for C_27_H_23_N_3_O_2_S: C, 71.50; H, 5.11; N, 9.26; found C, 71.67; H, 5.04; N, 9.51; HRMS *m*/*z* 454.15833 M^+^ + 1, calcd for C_27_H_23_N_3_O_2_S: 454.15837.

#### 4.1.3. General Procedure for the Preparation of Target Compounds **7a**–**d**

Route A:

A mixture of isatins **1c**, **e**–**g** (1 mmoL) with an equimolar amount of 2-mercaptobenzimidazole **6** (0.15 gm, 1 mmoL) and bromoacetic acid **3** (0.14 g, 1 mmoL) in glacial acetic acid (10 mL) in the presence of sodium acetate (0.16 g, 2 mmoL), was heated under reflux for 2 h. The obtained solid was filtered off while hot, washed with hot ethanol, dried and recrystallized from DMF to afford the target hybrids **7a**–**d**.

Route B:

To a stirred solution of 2-mercaptobenzimidazole **6** (0.15 gm, 1 mmoL) and KOH (0.06 gm, 1.1 mmoL) in ethanol (10 mL), bromoacetic acid **3** (0.14 gm, 1 mmoL) was added, and then the reaction mixture was refluxed for 1 h. The formed solid was filtered off, washed with ethanol to give compound **8**, which cyclized into intermediate **9** via heating with acetic anhydride in pyridine medium at 100 °C. Intermediate **9** (0.19 g, 1 mmoL) was added to a stirred solution of the appropriate isatin derivative **1c**, **e**–**g** (1 mmoL) and sodium acetate (0.16 g, 2 mmoL) in glacial acetic acid (5 mL), then the reaction mixture was refluxed for 2 h. The formed solid was filtered off, washed with ethanol and recrystallized from DMF to furnish compounds **7a**–**d**.

2-(5-Chloro-2-oxoindolin-3-ylidene)benzo[4,5]imidazo[2,1-b]thiazol-3(2*H*)-one (**7a**)

Red powder (yield 80%), m.p. > 300 °C; IR (KBr, *ν* cm^−1^): 3325 (NH) and 1689 (C=O); ^1^H-NMR (DMSO-*d*_6_, 400 MHz) *δ ppm*: 7.04 (d, *J* = 8.3 Hz, 1H), 7.36–7.48 (m, 1H), 7.52 (dd, *J* = 8.4, 2.0 Hz, 1H), 7.69 (d, *J* = 6.6 Hz, 1H), 8.03 (d, *J* = 6.5 Hz, 1H), 8.93 (d, *J* = 2.1 Hz, 1H), 11. 51 (s, 1H, NH isatin, D_2_O exchangeable); Anal. Calcd. for C_17_H_8_ClN_3_O_2_S: C, 57.72; H, 2.28; N, 11.88; found C, 58.06; H, 2.41; N, 12.16.

2-(5-Methoxy-2-oxoindolin-3-ylidene)benzo[4,5]imidazo[2,1-b]thiazol-3(2*H*)-one (**7b**)

Red powder (yield 85%), m.p. > 300 °C; IR (KBr, *ν* cm^−1^): 3296 (NH) and 1692 (C=O); ^1^H-NMR (DMSO-*d*_6_, 400 MHz) *δ ppm*: 3.83 (s, 3H, -OCH_3_), 6.93 (d, *J* = 8.5 Hz, 1H, Ar-H), 7.08 (dd, *J* = 8.5, 2.6 Hz, 1H, Ar-H), 7.42 (m, 2H, Ar-H), 7.69 (d, *J* = 7.0 Hz, 1H, Ar-H), 8.01 (d, *J* = 6.6 Hz, 1H, Ar-H), 8.59 (d, *J* = 2.6 Hz, 1H, Ar-H), 11.18 (s, 1H, NH isatin, D_2_O exchangeable); MS, *m/z* [%]: 349 [M^+^, 21.03]; Anal. Calcd. for C_18_H_11_N_3_O_3_S: C, 61.88; H, 3.17; N, 12.03; found C, 62.14; H, 3.42; N, 12.31.

2-(5-Methyl-2-oxoindolin-3-ylidene)benzo[4,5]imidazo[2,1-b]thiazol-3(2*H*)-one (**7c**)

Red powder (yield 87%), m.p. > 300 °C; IR (KBr, *ν* cm^−1^): 3405 (NH) and 1693 (C=O); ^1^H-NMR (DMSO-*d*_6_, 400 MHz) *δ ppm*: 2.37 (s, 3H, -CH_3_), 6.90 (d, *J* = 7.6 Hz, 1H, Ar-H), 7.27 (d, *J* = 8.5, 1H, Ar-H), 7.33–7.43 (m, 2H, Ar-H), 7.68 (d, *J* = 7.2 Hz, 1H, Ar-H), 8.01 (d, *J* = 7.6 Hz, 1H, Ar-H), 8.75 (s, 1H, Ar-H), 11.26 (s, 1H, NH isatin, D_2_O exchangeable); MS, *m/z* [%]: 333 [M^+^, 23.48]; Anal. Calcd. for C_18_H_11_N_3_O_2_S: C, 64.85; H, 3.33; N, 12.61; found C, 64.97; H, 3.50; N, 12.89.

2-(5,7-Dimethyl-2-oxoindolin-3-ylidene)benzo[4,5]imidazo[2,1-b]thiazol-3(2*H*)-one (**7d**)

Red powder (yield 88%), m.p. > 300 °C; IR (KBr, *ν* cm^−1^): 3382 (NH) and 1689 (C=O); ^1^H-NMR (DMSO-*d*_6_, 400 MHz) *δ ppm*: 2.20 (s, 3H, -CH_3_), 2.29 (s, 3H, -CH_3_), 7.09 (s, 1H, Ar-H), 7.34–7.48 (m, 2H, Ar-H), 7.68 (d, *J* = 7.0 Hz, 1H), 8.02 (d, *J* = 7.0 Hz, 1H), 8.62 (s, 1H, Ar-H), 11.28 (s, 1H, NH isatin, D_2_O exchangeable); MS, *m/z* [%]: 347 [M^+^, 100]; Anal. Calcd. for C_19_H_13_N_3_O_2_S: C, 65.69; H, 3.77; N, 12.10; found C, 65.91; H, 3.86; N, 11.97.

### 4.2. Biological Evaluation

#### 4.2.1. Antiproliferative Activity Against Breast Cancer Cell Lines

Breast cancer cell lines MDA-MB-231 and MCF-7 cells were obtained from VACSERA Tissue Culture Unit. The cells were propagated in DMEM supplemented with 10% heat-inactivated fetal bovine serum, 1% l-glutamine (2.5 mM), HEPES (4-(2-hydroxyethyl)-1-piperazineethanesulfonic acid) buffer (10 mM) and 50 µg/mL gentamycin. All cells were maintained at 37 °C in a humidified atmosphere with 5% CO_2_ and were sub-cultured two times a week. Cytotoxicity was determined following the Crystal Violet (CV) cell cytotoxicity assay [41].

Test samples were dissolved in DMSO and kept at a stock concentration of 100 mM. Exponentially growing cells were collected using 0.25% Trypsin-EDTA and seeding was done at a density of 3000 cells/well in 96-well plates. After 24 h, the monolayer cultured cells were washed with sterile phosphate buffered saline (0.01 M pH 7.2) and simultaneously the cells were treated with 100 mL from 10 different dilutions of the test compounds as well as Doxorubicin was used as a standard compound in fresh maintenance medium and incubated at 37 °C for 24 h. Control wells were exposed to the same concentration of vehicle, DMSO (1%) used in the highest concentration of the test samples. Cell viability was >99% compared to untreated cells. The number of the surviving cells was determined by staining the cells with crystal violet followed by cell lysing using 33% glacial acetic acid. The absorbance was measured at 490 nm using ELISA reader (Thermo Fisher Scientific, Waltham, MA, USA) after gentle shaking. The absorbance values from untreated cells were considered as 100% proliferation.

The number of viable cells was determined using ELISA reader as previously mentioned and the percentage of viability was calculated as [1−(ODt/ODc)] × 100% where ODt is the mean optical density of wells treated with the test sample and ODc is the mean optical density of untreated cells. The 50% inhibitory concentration (IC_50_) was estimated from graphic plots of the dose response curve for each conc. using GraphPad Prism software, Version 5 (San Diego, CA, USA). The data presented are the mean of at least three separate experiments.

#### 4.2.2. In Vitro Cytotoxic Activity towards Nontumorigenic Human WI-38 and MCF-10A Cells

The cytotoxic activity of the active conjugates towards nontumorigenic human lung fibroblast cell line (WI-38) and human breast epithelial cell line (MCF-10A) was determined using Crystal violet (CV) cell cytotoxicity assay according to the previously published procedures [42,55].

#### 4.2.3. ELISA Immunoassay

The levels of the apoptotic markers (Bax, caspase-3) as well as the antiapoptotic marker Bcl-2 were determined using ELISA colorimetric kits per the manufacturer’s instructions, as reported earlier [41]. MDA-MB-231 cells were cultured as a monolayer in T-25 flasks and were seeded to attain 30% confluency prior to treatment. Cells were then treated separately with compounds **4m** and **7b** at their IC_50_ concentrations for 48 h. At the end of treatment, cells were collected via trypsinization and centrifuged at 10,000 rpm. The pellet was then rinsed with PBS and lysed in RIPA lysis buffer at 4 °C for 45 min, then centrifuged at 14,000 rpm for 20 min to remove the cellular debris. Lysates were then collected and stored at −80 °C for later protein determination using Pierce BCA Protein Assay Kit (Pierce Biotechnology, Rockford, IL, USA) according to manufacturer’s recommendations.

The cell lysate was diluted 10 times, and 100 μL (50 mg protein) was added to the wells of four separate microtiter plates for the four ELISA kits that were pre-coated with primary antibodies specific to Bax, Bcl-2 and caspase-3 proteins, respectively. A secondary biotin-linked antibody specific to the protein captured by the primary antibody was further added to bind the captured protein, forming a “sandwich” of specific antibodies around the desired protein in the cell lysate. The streptavidin-HRP complex was then used to bind the biotin-linked secondary antibody through its streptavidin portion. The HRP domain reacted with the added TMB substrate to form a colored product that measured at 450 nm by a plate reader (ChroMate-4300, Orlando, FL, USA) after the reaction was terminated via the addition of stop solution.

#### 4.2.4. Cell Cycle Analysis

TNBC MDA-MB-231 cells were treated with compound **4m** for 24 h (at its IC_50_ concentration), and then cells were washed twice with ice-cold phosphate buffered saline (PBS). Subsequently, the treated cells were collected by centrifugation, fixed in ice-cold 70% (*v*/*v*) ethanol, washed with PBS, re-suspended with 100 μg/mL RNase, stained with 40 μg/mL PI, and analyzed by flow cytometry using FACS Calibur (Becton Dickinson, BD, Franklin Lakes, NJ, USA). The cell cycle distributions were calculated using CellQuest software 5.1 (Becton Dickinson) [35,49].

#### 4.2.5. Annexin V-FITC Apoptosis Assay

Phosphatidylserine externalization was assayed using Annexin V-FITC/PI apoptosis detection kit (BD Biosciences, San Jose, CA, USA) according to the manufacturer’s instructions, as reported earlier [35,49]. MDA-MB-231 cells were cultured to a monolayer then treated with conjugate **4m** at its IC_50_ concentration as described earlier. Cells were then harvested via trypsinization, and rinsed twice in PBS followed by binding buffer. Moreover, cells were re-suspended in 100 μL of binding buffer with the addition of 1 μL of FITC-Annexin V (Becton Dickinson BD Pharmingen^TM^, Heidelberg, Germany) followed by an incubation period of 30 min at 4 °C. Cells were then rinsed in binding buffer and resuspended in 150 μL of binding buffer with the addition of 1 μL of DAPI (1 μg/μL in PBS) (Invitrogen, Life Technologies, Darmstadt, Germany). Cells were then analyzed using the flow cytometer BD FACS Canto II (BD Biosciences, San Jose, CA, USA) and the results were interpreted with FlowJo7.6.4 software (Tree Star, Ashland, OR, USA).

#### 4.2.6. Statistical Analysis

Data are presented as means ± S.D. Individual groups were compared using the two-tailed independent Student’s *t*-test. Multiple group comparisons were carried out using one-way analysis of variance (ANOVA) followed by the Tukey–Kramer test for post-hoc analysis. Statistical significance was accepted at a level of *p* < 0.05. All statistical analyses were performed using GraphPad InStat software, version 3.05 (GraphPad Software, Inc., La Jolla, CA, USA). Graphs were sketched using GraphPad Prism software, version 5.00 (GraphPad Software, Inc., La Jolla, CA, USA).

## Figures and Tables

**Table 1:**
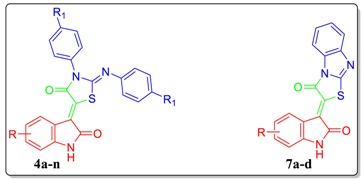
In vitro antiproliferative activity of hybrids **4a**–**n** and **7a**–**d** against MDA-MB-231 and MCF-7 breast cancer cell lines.

Compound	R	R_1_	IC_50_ (µM) ^a^	
			MDA-MB-231	MCF-7
**4a**	H	H	NA ^b^	NA ^b^
**4b**	5-F	H	108.3 ± 8.2	58.5 ± 3.2
**4c**	5-Cl	H	24.1 ± 2.1	29.2 ± 2.5
**4d**	5-Br	H	15.7 ± 0.9	17.1 ± 1.1
**4e**	5-OCH_3_	H	42.6 ± 3.4	52.9 ± 5.0
**4f**	5-CH_3_	H	60.9 ± 4.2	22.8 ± 2.0
**4g**	5,7-(CH_3_)_2_	H	43.6 ± 3.1	47.4 ± 3.9
**4h**	H	CH_3_	51.7 ± 2.1	56.0 ± 3.7
**4i**	5-F	CH_3_	152.1 ± 11.5	104.9 ± 7.5
**4j**	5-Cl	CH_3_	50.8 ± 3.5	NA ^b^
**4k**	5-Br	CH_3_	NA ^b^	NA ^b^
**4l**	5-OCH_3_	CH_3_	47.1 ± 4.1	53.5 ± 3.6
**4m**	5-CH_3_	CH_3_	7.6 ± 0.5	8.4 ± 0.5
**4n**	5,7-(CH_3_)_2_	CH_3_	36.3 ± 2.9	23.7 ± 1.9
**7a**	5-Cl	-	47.2 ± 3.8	127.3 ± 9.9
**7b**	5-OCH3	-	13.2 ± 1.7	21.7 ± 1.5
**7c**	5-CH3	-	40.3 ± 2.5	48.2 ± 2.7
**7d**	5,7-(CH_3_)_2_	-	23.5 ± 1.0	27.6 ± 2.4
**Dox.**			4.7 ± 0.4	3.8 ± 0.4
**Sunitinib**			5.5 ± 0.5	3.4 ± 0.3

^a^ IC_50_ values are the mean ± S.D. of three separate experiments; ^b^ NA: Compounds having IC_50_ value > 200 µM.

**Table 2:** In vitro cytotoxic activity against nontumorigenic cell lines (WI-38 and MCF-10A), and selectivity index (MCF-10A/ MDA-MB-231) for the active compounds.

Compound	IC_50_ (µM) ^a^			Selectivity Index
	Lung WI-38	Breast MCF-10A	MDA-MB-231	
**4c**	87.2 ± 5.2	112.6 ± 5.9	24.1 ± 2.1	4.7
**4d**	65.5 ± 4.2	80.7 ± 3.4	15.7 ± 0.9	5.1
**4e**	193.1 ± 10.6	89.6 ± 5.1	42.6 ± 3.4	2.1
**4g**	120.0 ± 7.8	141.7 ± 7.9	43.6 ± 3.1	3.25
**4l**	176.2 ± 11.3	157.4 ± 6.8	47.2 ± 4.1	3.3
**4m**	49.1 ± 1.9	73.1 ± 2.7	7.6 ± 0.5	9.6
**4n**	218.3 ± 13.5	154.0 ± 5.2	36.3 ± 2.9	4.2
**7a**	353.0 ± 17.1	233.8 ± 9.6	47.2 ± 3.8	5.0
**7b**	121.1 ± 6.9	183.9 ± 8.0	13.2 ± 1.1	13.9
**7c**	192.0 ± 10.8	242.2 ± 11.5	40.3 ± 2.5	6.0
**7d**	234.0 ± 15.4	250.3 ± 9.8	23.5 ± 1.00	10.7

^a^ IC_50_ values are the mean ± S.D. of three separate experiments.

**Table 3:** Impact of hybrids **4m** and **7b** on the expression levels of Bcl-2 and Bax and on the active caspase-3 level in MDA-MB-231 cancer cells treated with each hybrid at its IC_50_ concentration.

Compound	Bax (pg/mg of Total Protein)	Bcl-2 (ng/mg of Total Protein)	Bax/Bcl-2	Caspase-3 (ng/mg of Total Protein)
**4m**	274.8 ± 13.5 (11.1) *	2.1 ± 0.1 (0.33) *	130.5	0.19 ± 0.01 (6.4) *
**7b**	180.0 ± 7.3 (7.3) *	2.8 ± 0.1 (0.45) *	64.1	0.15 ± 0.01 (4.9) *
**Control**	24.7 ± 1.7	6.3 ± 0.2	3.9	0.03 ± 0.01

* Numbers given between parentheses are the numbers of folds of control.

**Table 4:** Computer-aided ADME study for the synthesized conjugates **4a**–**m** and **7a**–**d.**

Compound	AlogP98 ^a^	PSA_2D ^b^	Solubility ^c^	Solubility Level ^d^	Absorption Level ^e^	CYP2D6 ^f^
**4a**	4.476	62.087	−6.305	1	0	0
**4b**	4.682	62.087	−6.605	1	0	0
**4c**	5.141	62.087	−7.005	1	0	0
**4d**	5.225	62.087	−7.079	1	0	0
**4e**	4.46	71.017	−6.292	1	0	0
**4f**	4.962	62.087	−6.765	1	0	0
**4g**	5.449	62.087	−7.226	1	0	0
**4h**	5.449	62.087	−7.197	1	0	0
**4i**	5.654	62.087	−7.484	1	1	0
**4j**	6.113	62.087	−7.885	1	1	0
**4k**	6.197	62.087	−7.958	1	1	0
**4l**	5.432	71.017	−7.159	1	0	0
**4m**	5.935	62.087	−7.645	1	1	0
**4n**	6.421	62.087	−8.093	0	1	0
**7a**	3.581	64.021	−6.005	1	0	0
**7b**	2.9	72.951	−5.327	2	0	0
**7c**	3.403	64.021	−5.771	2	0	0
**7d**	3.889	64.021	−6.263	1	0	0

^a^ Lipophilicity descriptor. ^b^ Polar surface area. ^c^ Solubility parameter.(0:−2 = optimal, −2:−4 = good, −4:−6 = low, −6:−8 = very low). ^d^ Solubility level. (0 = extremely low, 1 = very low but possible, 2 = low, 3 = good, 4 = optimal). ^e^ Absorption level. (0 = good, 1 = moderate, 2 = low, 3 = very low). ^f^ CYP2D6 inhibition. (0 = noninhibitor, 1 = inhibitor).

## References

[1] DeSantis C., Ma J., Bryan L., Jemal A. (2014). Breast cancer statistics, 2013. CA Cancer J. Clin..

[2] International Agency for Research on Cancer (2014). IGlobocan: Estimated Cancer Incidence, Mortality, and Prevalence Worldwide in 2012.

[3] Siegel R., Ma J., Zou Z., Jemal A. (2014). Cancer statistics, 2014. CA Cancer J. Clin..

[4] Adami H., Hunter D., Trichopoulos D. (2002). Textbook of Cancer Epidemiology.

[5] Barnard M.E., Boeke C.E., Tamimi R.M. (2015). Established breast cancer risk factors and risk of intrinsic tumor subtypes. Biochim. Biophys. Acta.

[6] Rahman M., Davis S.R., Pumphrey J.G., Bao J., Nau M.M., Meltzer P.S., Lipkowitz S. (2009). TRAIL induces apoptosis in triple-negative breast cancer cells with a mesenchymal phenotype. Breast Cancer Res. Treat..

[7] Abdel-Aziz H.A., Eldehna W.M., Ghabbour H., Al-Ansary G.H., Assaf A.M., Al-Dhfyan A. (2016). Synthesis, crystal study, and antiproliferative activity of some 2-benzimidazolylthioacetophenones towards triple-negative breast cancer MDA-MB-468 cells as apoptosis-inducing agents. Int. J. Mol. Sci..

[8] Smid M., Wang Y., Zhang Y., Sieuwerts A.M., Yu J., Klijn J.G., Foekens J.A., Martens J.W. (2008). Subtypes of breast cancer show preferential site of relapse. Cancer Res..

[9] Medvedev A., Buneeva O., Gnedenko O., Ershov P., Ivanov A. (2018). Isatin, an endogenous nonpeptide biofactor: A review of its molecular targets, mechanisms of actions, and their biomedical implications. BioFactors.

[10] Kidwai M., Jahan A., Mishra N.K. (2014). Isatins: A diversity orientated biological profile. Med. Chem..

[11] Eldehna W.M., Al-Ansary G.H., Bua S., Nocentini A., Gratteri P., Altoukhy A., Ghabbour H., Ahmed H.Y., Supuran C.T. (2017). Novel indolin-2-onebased sulfonamides as carbonic anhydrase inhibitors: Synthesis, in vitro biological evaluation against carbonic anhydrases isoforms I, II, IV and VII and molecular docking studies. Eur. J. Med. Chem..

[12] Eldehna W.M., Fares M., Abdel-Aziz M.M., Abdel-Aziz H.A. (2015). Design, synthesis and antitubercular activity of certain nicotinic Acid hydrazides. Molecules.

[13] Sridhar S.K., Ramesh A. (2001). Synthesis and pharmacological activities of hydrazones, Schiff and Mannich bases of isatin derivatives. Biol. Pharm. Bull..

[14] Vine K.L., Matesic L., Locke J.M., Skropeta D. (2013). Recent highlights in the development of isatin-based anticancer agents. Adv. Anticancer Agents Med. Chem..

[15] Goodman V.L., Rock E.P., Dagher R., Ramchandani R.P., Abraham S., Gobburu J.V., Booth B.P., Verbois S.L., Morse D.E., Liang C.Y. (2007). Approval summary: Sunitinib for the treatment of imatinib refractory or intolerant gastrointestinal stromal tumors and advanced renal cell carcinoma. Clin. Cancer Res..

[16] McCormack P.L. (2015). Nintedanib: First global approval. Drugs.

[17] Lemmens L. (2016). Nintedanib in advanced NSCLC: Management of adverse events. Lung Cancer Manag..

[18] Nagarsenkar A., Guntuku L., Guggilapu S.D., Gannoju S., Naidu V.G.M., Bathini N.B. (2016). Synthesis and apoptosis inducing studies of triazole linked 3-benzylidene isatin derivatives. Eur. J. Med. Chem..

[19] Karthikeyan C., Solomon V.R., Lee H., Trivedi P. (2013). Design, synthesis and biological evaluation of some isatin-linked chalcones as novel anti-breast cancer agents: A molecular hybridization approach. Biomed. Prev. Nutr..

[20] Ke S., Shi L., Yang Z. (2015). Discovery of novel isatin–dehydroepiandrosterone conjugates as potential anticancer agents. Bioorg. Med. Chem. Lett..

[21] Solomon V.R., Hu C., Lee H. (2009). Hybrid pharmacophore design and synthesis of isatin–benzothiazole analogs for their anti-breast cancer activity. Bioorg. Med. Chem..

[22] Abdel-Aziz H.A., Elsaman T., Al-Dhfyan A., Attia M.I., Al-Rashood K.A., Al-Obaid A.M. (2013). Synthesis and Anticancer Potential of Certain Novel 2-Oxo-*N*′-(2-oxoindolin-3-ylidene)-2*H*-chromene-3-carbohydrazides. Eur. J. Med. Chem..

[23] Gudipati R., Anreddy R.N.R., Manda S. (2011). Synthesis, anticancer and antioxidant activities of some novel *N*-(benzo[d]oxazol-2-yl)-2-(7- or 5-substituted-2-oxoindolin-3-ylidene) hydrazinecarboxamide derivatives. J. Enzyme Inhib. Med. Chem..

[24] Aboul-Fadl T., Kadi A., Abdel-Aziz H.A. (2012). Novel *N*,*N*′-Hydrazino-bis-isatin Derivatives with Selective Activity Against Multidrug-Resistant Cancer Cells. U.S. Patent.

[25] Havrylyuk D., Kovach N., Zimenkovsky B., Vasylenko O., Lesyk R. (2011). Synthesis and Anticancer Activity of Isatin-Based Pyrazolines and Thiazolidines Conjugates. Arch. Pharm. Chem. Life Sci..

[26] Ramshid P.K., Jagadeeshan S., Krishnan A., Mathew M., Nair S.A., Pillai M.R. (2010). Synthesis and in vitro Evaluation of Some Isatin-Thiazolidinone Hybrid Analogues as Anti-Proliferative Agents. Med. Chem..

[27] Kaminskyy D., Khyluk D., Vasylenko O., Zaprutko L., Lesky R. (2011). A Facile Synthesis and Anticancer Activity Evaluation of Spiro[Thiazolidinone-Isatin] Conjugates. Sci. Pharm..

[28] Havrylyuk D., Zimenkovsky B., Vasylenko O., Gzella A., Lesyk R. (2012). Synthesis of New 4-Thiazolidinone-, Pyrazoline-, and Isatin-Based Conjugates with Promising Antitumor Activity. J. Med. Chem..

[29] Wang S., Zhao Y., Zhang G., Lv Y., Zhang N., Gong P. (2011). Design, synthesis and biological evaluation of novel 4-thiazolidinones containing indolin-2-one moiety as potential antitumor agent. Eur. J. Med. Chem..

[30] Taher A.T., Khalil N.A., Ahmed E.M. (2011). Synthesis of Novel Isatin-Thiazoline and Isatin-Benzimidazole Conjugates as Anti-Breast Cancer Agents. Arch. Pharm. Res..

[31] Eldehna W.M., Almahli H., Al-Ansary G.H., Ghabbour H.A., Aly M.H., Ismael O.E., Al-Dhfyan A., Abdel-Aziz H.A. (2017). Synthesis and in vitro antiproliferative activity of some novel isatins conjugated with quinazoline/phthalazine hydrazines against triple-negative breast cancer MDA-MB-231 cells as apoptosis-inducing agents. J. Enz. Inhib. Med. Chem..

[32] Abdel-Aziz H.A., Eldehna W.M., Keeton A.B., Piazza G.A., Kadi A.A., Attwa M.W., Abdelhameed A.S., Attia M.I. (2017). Isatin-benzoazine molecular hybrids as potential antiproliferative agents: Synthesis and in vitro pharmacological profiling. Drug Des. Dev. Ther..

[33] Eldehna W.M., Fares M., Ibrahim H.S., Alsherbiny M.A., Aly M.H., Ghabbour H.A., Abdel-Aziz H.A. (2016). Synthesis and cytotoxic activity of biphenylurea derivatives containing indolin-2-one moieties. Molecules.

[34] Eldehna W.M., EL-Naggar D.H., Hamed A.R., Ibrahim H.S., Ghabbour H.A., Abdel-Aziz H.A. (2018). One-pot three-component synthesis of novel spirooxindoles with potential cytotoxic activity against triple-negative breast cancer MDA-MB-231 cells. J. Enz. Inhib. Med. Chem..

[35] Eldehna W.M., Abo-Ashour M.F., Ibrahim H.S., Al-Ansary G.H., Ghabbour H.A., Elaasser M.M., Ahmed H.Y., Safwat N.A. (2018). Novel [(3-indolylmethylene)hydrazono]indolin-2-ones as apoptotic antiproliferativeagents: Design, synthesis and in vitro biological evaluation. J. Enz. Inhib. Med. Chem..

[36] Attia M.I., Eldehna W.M., Afifi S.A., Keeton A.B., Piazza G.A., Abdel-Aziz H.A. (2017). New hydrazonoindolin-2-ones: Synthesis, exploration of the possible antiproliferative mechanism of action and encapsulation into PLGA microspheres. PLoS ONE.

[37] Eldehna W.M., Altoukhy A., Mahrous H., Abdel-Aziz H.A. (2015). Design, synthesis and QSAR study of certain isatin-pyridine hybrids as potential antiproliferative agents. Eur. J. Med. Chem..

[38] Abdel-Aziz H.A., Ghabbour H.A., Eldehna W.M., Qabeel M.M., Fun H.-K. (2014). Synthesis, crystal structure and biological activity of *cis*/*trans* amide rotomers of (*Z*)-*N*′-(2-oxoindolin-3-ylidene)formohydrazide. J. Chem..

[39] Eldehna W.M., Fares M., Ceruso M., Ghabbour H.A., Abou-Seri S.M., Abdel-Aziz H.A., El Ella D.A.A., Supuran C.T. (2016). Amido/ureidosubstituted benzenesulfonamides-isatin conjugates as low nanomolar/subnanomolar inhibitors of the tumor-associated carbonic anhydrase isoform XII. Eur. J. Med. Chem..

[40] Eldehna W.M., Fares M., Ibrahim H.S., Aly M.H., Zada S., Ali M.M., Abou-Seri S.M., Abdel-Aziz H.A., El Ella D.A.A. (2015). Indoline ureas as potential anti-hepatocellular carcinoma agents targeting VEGFR-2: Synthesis, in vitro biological evaluation and molecular docking. Eur. J. Med. Chem..

[41] Eldehna W.M., Abo-Ashour M.F., Nocentini A., Gratteri P., Eissa I.H., Fares M., Ismael O.E., Ghabbour H.A., Elaasser M.M., Abdel-Aziz H.A. (2017). Novel 4/3-((4-oxo-5-(2-oxoindolin-3-ylidene) thiazolidin-2-ylidene) amino) benzenesulfonamides: Synthesis, carbonic anhydrase inhibitory activity, anticancer activity and molecular modelling studies. Eur. J. Med. Chem..

[42] Eldehna W.M., Al-Wabli R.I., Almutairi M.S., Keeton A.B., Piazza G.A., Abdel-Aziz H.A., Attia M.I. (2018). Synthesis and biological evaluation of certain hydrazonoindolin-2-one derivatives as new potent antiproliferative agents. J. Enz. Inhib. Med. Chem..

[43] Delbridge A.R., Grabow S., Strasser A., Vaux D.L. (2016). Thirty years of BCL-2: Translating cell death discoveries into novel cancer therapies. Nat. Rev. Cancer.

[44] Hu W., Kavanagh J.J. (2003). Anticancer therapy targeting the apoptotic pathway. Lancet Oncol..

[45] Lopez J., Tait S.W.G. (2015). Mitochondrial apoptosis: Killing cancer using the enemy within. Br. J. Cancer.

[46] Youle R.J., Strasser A. (2008). The BCL-2 protein family: Opposing activities that mediate cell death. Nat. Rev. Mol. Cell Biol..

[47] Jiang H., Zhao P.J., Su D., Feng J., Ma S.L. (2014). Paris saponin I induces apoptosis via increasing the Bax/Bcl-2 ratio and caspase-3 expression in gefitinib-resistant non-small cell lung cancer in vitro and in vivo. Mol. Med. Rep..

[48] McIlwain D.R., Berger T., Mak T.W. (2015). Caspase functions in cell death and disease. Cold Spring Harb. Perspect. Biol..

[49] Eldehna W.M., Ibrahim H.S., Abdel-Aziz H.A., Farrag N.N., Youssef M.M. (2015). Design, synthesis and in vitro antitumor activity of novel *N*-substituted-4-phenyl/benzylphthalazin-1-ones. Eur. J. Med. Chem..

[50] Accelrys Co. Ltd. Discovery Studio 4.0. http://www.accelrys.com/.

[51] Fares M., Said M.A., Alsherbiny M.A., Eladwy R.A., Almahli H., Abdel-Aziz M.M., Ghabbour H.A., Eldehna W.M., Abdel-Aziz H.A. (2016). Synthesis, biological evaluation and molecular docking of certain sulfones as potential nonazole antifungal agents. Molecules.

[52] Meng G., Zheng M., Wang M., Tong J., Ge W., Zhang J., Zheng A., Li J., Gao L., Li J. (2016). Design and synthesis of new potent PTP1B inhibitors with the skeleton of 2-substituted imino-3-substituted-5-heteroarylidene-1, 3-thiazolidine-4-one: Part I. Eur. J. Med. Chem..

[53] Mavrova A.T., Anichina K.K., Vuchev D.I., Tsenov J.A., Denkova P.S., Kondeva M.S., Micheva M.K. (2006). Antihelminthic activity of some newly synthesized 5 (6)-(un) substituted-1H-benzimidazol-2-ylthioacetylpiperazine derivatives. Eur. J. Med. Chem..

[54] Hu J., Wang Y., Wei X., Wu X., Chen G., Cao G., Shen X., Zhang X., Tang Q., Liang G. (2013). Synthesis and biological evaluation of novel thiazolidinone derivatives as potential anti-inflammatory agents. Eur. J. Med. Chem..

[55] Soliman D.H., Eldehna W.M., Ghabbour H.A., Kabil M.M., Abdel-Aziz M.M., Abdel-Aziz H.A.K. (2017). Novel 6-Phenylnicotinohydrazide Derivatives: Design, Synthesis and Biological Evaluation as a Novel Class of Antitubercular and Antimicrobial Agents. Biol. Pharm. Bull..

